# Short-, Mid-, and Long-Term Effect of Granulocyte Colony-Stimulating Factor/Stem Cell Factor and Fms-Related Tyrosine Kinase 3 Ligand Evaluated in an In Vivo Model of Hypoxic-Hyperoxic Ischemic Neonatal Brain Injury

**DOI:** 10.1155/2019/5935279

**Published:** 2019-03-13

**Authors:** Anna Posod, Karina Wegleiter, Vera Neubauer, Martina Urbanek, Eva Huber, Ursula Kiechl-Kohlendorfer, Matthias Keller, Elke Griesmaier

**Affiliations:** ^1^Department of Pediatrics II (Neonatology), Medical University of Innsbruck, Anichstrasse 35, 6020 Innsbruck, Austria; ^2^Kinderklinik Dritter Orden, Munich Technical University, Bischof Altmann-Strasse 9, 94032 Passau, Germany

## Abstract

Hematopoietic growth factors are considered to bear neuroprotective potential. We have previously shown that delayed treatment with granulocyte colony-stimulating factor (G-CSF)/stem cell factor (SCF) and Fms-related tyrosine kinase 3 ligand (FL) ameliorates excitotoxic neonatal brain injury. The effect of these substances in combined-stressor neonatal brain injury models more closely mimicking clinical conditions has not been investigated. The aim of this study was to assess the short-, mid-, and long-term neuroprotective potential of G-CSF/SCF and FL in a neonatal model of hypoxic-hyperoxic ischemic brain injury. Five-day-old (P5) CD-1 mice were subjected to unilateral common carotid artery ligation and subsequent alternating periods of hypoxia and hyperoxia for 65 minutes. Sixty hours after injury, pups were randomly assigned to intraperitoneal treatment with (i) G-CSF (200 *μ*g/kg)/SCF (50 *μ*g/kg), (ii) FL (100 *μ*g/kg), or (iii) vehicle every 24 hours for three or five consecutive days. Histopathological and functional outcomes were evaluated on P10, P18, and P90. Baseline outcome parameters were established in sham-treated and healthy control animals. Gross brain injury did not significantly differ between treatment groups at any time point. On P10, caspase-3 activation and caspase-independent apoptosis were similar between treatment groups; cell proliferation and the number of BrdU-positive vessels did not differ on P18 or P90. Neurobehavioral assessment did not reveal significant differences between treatment groups in accelerod performance, open field behavior, or novel object recognition capacity on P90. Turning behavior was more frequently observed in G-CSF/SCF- and FL-treated animals. No sex-specific differences were detected in any outcome parameter evaluated. In hypoxic-hyperoxic ischemic neonatal brain injury, G-CSF/SCF and FL treatment does not convey neuroprotection. Prior to potential clinical use, meticulous assessment of these hematopoietic growth factors is mandated.

## 1. Introduction

Neonatal brain injury is a problem of great global concern [1, 2]. Advances in perinatal care have improved survival rates of affected infants, but long-term morbidity is still substantial [3, 4]. Etiopathogenesis is complex and incompletely understood, and various mechanisms are thought to contribute, including excitotoxicity, hypoxia-ischemia, inflammation, and additional factors such as oxidative stress/hyperoxia [5–7]. To date, causal pharmacologic therapies are not available for routine clinical use. The most auspicious treatment options are those that intervene on multiple levels and are able to not only inhibit cell death and promote cell survival, but also foster regeneration and functional reorganisation [8–10]. Interventions augmenting endogenous neuroprotective responses, among them cytokines and growth factors, hold particular promise [11–14]. Hematopoietic growth factors have been extensively studied and used in neonates for hematologic indications for several years [15]. In addition to their hematopoietic effects, they may have other potential benefits, including neuroprotection, neural tissue repair, and neurovasculogenesis [15–17]. The effects of these agents on neuronal death or survival, however, seem to be context-specific [12], conveying neuroprotection in some neonatal brain injury models [18–28], while having no effect or even exacerbating damage in others [29, 30].

Our research group has recently been able to show that systemic treatment with the hematopoietic growth factors/cytokines granulocyte colony-stimulating factor (G-CSF)/stem cell factor (SCF) and Fms-related tyrosine kinase 3 ligand (FL) starting 60 hours after insult protects against N-methyl-D-aspartate receptor-mediated developmental excitotoxic brain damage by reducing injury extent and apoptotic cell death [31]. Whether these treatment regimina are effective and safe in other injury models, especially those encompassing multiple stressors, has not yet been investigated. Prior to clinical use, however, thorough assessment of treatment options in combined-stressor brain injury models is of utmost importance, as these may more adequately reflect and mimic the conditions neonates experience in intensive care units [32].

Furthermore, any neuromodulatory intervention taking place during potentially critical or sensitive periods of brain development possibly has not only short-, but also long-term impact [33]. The assessment of behavioral endpoints is thus crucial in order to evaluate whether morphological effects translate into differences in functional outcome [20].

Thus, the aim of the current study was to investigate short-, mid-, and long-term effects of delayed treatment with G-CSF/SCF and FL in a mouse model of combined hypoxic-hyperoxic ischemic neonatal brain injury. From our previous findings we hypothesised that G-CSF/SCF and FL reduce injury extent and apoptotic cell death, stimulate neuroproliferation, and improve neurobehavioral outcome following hypoxia-hyperoxia ischemia.

## 2. Materials and Methods

### 2.1. Materials

Heparin (Heparin Immuno 1000 IU/ml) was obtained from Ebewe Pharma (Unterach, Austria), and phenobarbital (Luminal®) was obtained from Desitin Arzneimittel GmbH (Hamburg, Germany); murine G-CSF, SCF, and FL were obtained from Peprotech (London, England), and 5-bromo-2′-deoxyuridine (BrdU) was obtained from Sigma-Aldrich (Vienna, Austria).

### 2.2. Animal Experiments

All animal studies were conducted in compliance with current EU legislation (Directive 2010/63/EU revising Directive 86/609/EEC) and Austrian law. Permission for this study was obtained by the ethics committee of the Austrian Federal Ministry of Education, Science and Research (BMWF-66.011/0131-II/10b/2008). CD-1 mice (Crl:CD1 (ICR), Charles River Laboratories, Sulzfeld, Germany) were bred and kept at the Central Laboratory Animal Facility, Medical University of Innsbruck, Austria, under standard housing conditions with a 12-hour light-dark cycle, a room temperature of 25°C, and a humidity of 75%. Five-day-old (P5) CD-1 mice of both sexes with a body weight (bw) of 3 ± 0.3 g were eligible for the study. For all experiments humane endpoints were considered as follows: Animals were controlled daily for pathological and behavioral changes, including exploratory activity, thirst, and drinking. Following the experimental procedures daily inspection of the animals was performed by trained personnel to detect signs of infection, pain, or discomfort. Abnormal feeding behavior, abnormal body performance, or isolation of animals was reported. If any sign was positive, reevaluation was performed the same day and if persistent a veterinary was asked to further evaluate the animal. Protocol included that, in case animals reached endpoint criteria, euthanasia would be performed immediately. Hypoxic-hyperoxic ischemic (HHI) brain injury was induced as described previously [34]. In brief, mouse pups were subjected to right common carotid artery ligation under general anesthesia (isoflurane in oxygen, 3.0 vol% induction/1.5 vol% maintenance; AbbVie, Vienna, Austria). After a 120-minute recovery period, they were exposed to 8% oxygen/nitrogen for 15 minutes, alternating with 100% oxygen for ten minutes for a total duration of 65 minutes in an incubated chamber kept at a constant ambient temperature of 34°C. Sixty hours after injury, pups were randomly assigned to one of the following treatment groups: (i) G-CSF (200 *μ*g/kg bw)/SCF (50 *μ*g/kg bw) in vehicle (1x PBS, phosphate-buffered saline), (ii) FL (100 *μ*g/kg bw) in vehicle, or (iii) vehicle only and received an intraperitoneal (i.p.) injection every 24 hours for three or five consecutive days, depending on timing of endpoint determination. Endpoints were assessed on P10, P18, and P90. Sex was visually determined by assessment of the anogenital distance and confirmed by polymerase chain reaction (P10, presence or absence of the SRY gene) or was determined by visual assessment alone (P18, P90) depending on mouse age as described previously [35]. Animals randomised to mid- (P18) and long-term endpoints (P90) received additional i.p. injections of BrdU solution (25 mg/kg bw) every 24 hours from P12 to P28. On P10, animals were sacrificed by decapitation; brains were harvested and immersion-fixed in 4% formaldehyde for 72-120 hours. On P18 and P90, animals were deeply anaesthetised with phenobarbital (200 mg/kg i.v.) and intracardially perfused with 0.9% saline solution containing 1000 IU heparin per 100 ml. To ensure optimal fixation, the perfusion solution was switched to 4% paraformaldehyde after clearing of blood. Brains were harvested and immediately fixed in 4% paraformaldehyde for 24 hours. Following paraffin embedding, all brains were cut into 10-*μ*m-thick coronal sections. Sham-treated animals underwent anesthesia and surgical preparation of the right common carotid artery without ligation; healthy control animals did not undergo any form of treatment.

### 2.3. Gross Brain Injury Assessment

Extent of brain injury was determined in cresyl violet-stained sections by a blinded observer as described previously [34]. For cresyl violet staining, 10 *μ*m-thick paraffin-embedded tissue sections were processed using an automatic staining device (Varistain 24-4 Automatic Slide Stainer; Thermo Fisher Scientific, MA, USA). Slides were deparaffinized using several changes of xylene, passed through graded alcohols, and then stained using 1% cresyl violet acetate (Sigma C5042; Sigma-Aldrich, Vienna, Austria) for 10 minutes. After differentiation in 3% acetic acid solution for 8 seconds and rapid dehydration with graded alcohols, slides were cleared with xylene and cover-slipped using PERTEX® mounting medium (VWR International, Darmstadt, Germany). Gross brain injury was assessed on a 5-point ordinal scale (from 0 to 4) as follows: 0=normal ipsilateral hemisphere; 1=mild edema/atrophy with <25% lesion of the ipsilateral hemisphere; 2=moderate atrophy with 25%-50% lesion; 3=severe atrophy with cystic cavitation of 50%-75%, and 4=severe atrophy with cystic cavitation >75%.

In addition, region-specific neuropathologic injury extent was determined in every section by two blinded observers according to a modified scoring system as follows: injury in cerebral cortex and adjacent white matter was scored 0–4 (0, no injury; 1, few small isolated groups of injured cells; 2, several larger groups of injured cells; 3, moderate confluent infarction; 4, extensive confluent infarction) and in hippocampus, striatum, and thalamus was scored 0–3 for mild, moderate, or severe atrophy or neuronal injury/infarction [29, 36]. The average of the scores of the two investigators was used for statistical analysis.

### 2.4. Immunohistochemical Analyses

Immunohistochemical slides were pretreated as published [37]. In brief, paraffin-embedded brain sections were deparaffinized and rehydrated through graded alcohols. Endogenous peroxidase activity was quenched by incubation with 2% hydrogen peroxide in methanol for 30 minutes. Heat-induced antigen retrieval was performed using citrate buffer. After nonspecific blocking with 1% bovine serum albumin (biotin-free) in TBS/0.05% Tween-20, sections were incubated overnight (4°C) with rabbit polyclonal anti-cleaved caspase-3 (Asp175) antibody (1:250, Cat. No. #9661, Cell Signaling Technology Europe, Frankfurt/Main, Germany), goat anti-apoptosis-inducing factor (AIF) antibody (1:300, Cat. No. sc-9416, Santa Cruz Biotechnologies, Heidelberg, Germany), or rat monoclonal anti-BrdU [BU1/75(ICR1)] antibody (1:50, Cat. No. ab6326, Abcam, Cambridge, UK). After rinsing with PBS, sections were incubated for 45-60 minutes at 25°C with the appropriate biotinylated secondary antibodies, namely, goat anti-rabbit IgG antibody (1:200, Cat. No. 111-065-003, Jackson ImmunoResearch, Cambridgeshire, UK), donkey anti-goat IgG (1:200, Cat. No. 705-065-003, Jackson ImmunoResearch), or goat anti-rat IgG antibody (1:100, Cat. No. BA-9400, Vector Laboratories, Burlingame, USA). Visualisation was performed by incubating with streptavidin-biotin complex (VECTASTAIN Elite ABC Kit, Cat. No. PK-6100, Vector Laboratories) for 45 minutes at 25°C using diaminobenzidine (Invitrogen, Fisher Scientific Austria, Vienna, Austria) as a chromogen. After multiple rinses in distilled water, sections were dehydrated, cleared in xylene, and cover-slipped. The Allen Mouse Brain Coronal Atlas (available from http://brain-map.org) was used as an anatomical reference for brain structures. Immunohistochemical quantification was conducted in different section planes depending on the respective regions of interest as follows: For counting of activated caspase-3 and AIF-positive nuclei, coronal levels 53 (Bregma 0.145 mm; striatum), 71 (Bregma -1.655 mm; thalamus), and 81 (Bregma -2.780 mm; hippocampus) were used as a reference. For BrdU, coronal levels 53 (Bregma 0.145 mm; subventricular zone, SVZ) and 81 (Bregma -2.780 mm; field CA1 of the hippocampus, CA1; granular layer of the hippocampal dentate gyrus, GrDG) were used as a reference. An Olympus IX83 microscope equipped with a DP27 digital color camera system and cellSens Dimension software (Olympus Austria, Vienna) was used to acquire images and count cells. ROIs were chosen ipsi- and contralateral for cortical grey matter, white matter, hippocampus, striatum, and thalamus.

Manual cell counting for activated caspase 3 and AIF-positive nuclei was performed in four adjacent sections per slide by a blinded observer in grey matter, underlying white matter, hippocampus, striatum, and thalamus at the above-mentioned stereotaxic coordinates at a 200x magnification within an area of 0.139 mm2 (one visual field). With regard to grey matter, cells were counted in cerebral cortex in 15 visual fields. In hippocampus, striatum, and thalamus, cells were counted in the entire displayed region. For quantification of BrdU-positive cells, the granular layer of dentate gyrus and CA1 region of the hippocampus were evaluated separately. BrdU-positive cells in the SVZ were counted in 3-4 cell diameters adjacent to the ependyma. Cell counts give absolute numbers of cells in the respective brain areas. For regions represented in more than one section plane (grey matter, white matter), mean values were calculated.

### 2.5. Behavioral Testing

Behavioral testing was conducted as described previously in detail [29]. At eleven weeks of age, animals were moved to a testing facility with a 12-hour light-dark cycle with lights on from 7 a.m. to 7 p.m. All tests were performed in accordance with circadian rhythms. Three different methods were used: (i) rotarod-accelerod to assess neuromotor performance, (ii) open field testing to observe general motor activity, exploratory behavior, and anxiety, and (iii) novel object recognition (NOR) to study learning and memory. Baseline behavioral parameters were assessed in healthy control animals for each task.

#### 2.5.1. Rotarod/Accelerod

For rotarod testing, a computer-aided system (TSE Systems, Bad Homburg, Germany) with an accelerating rod setting (accelerod-acceleration from 5 to 60 rpm over a five-minute period) was used. Animals were trained three times a day with a test-free interval of one hour on five consecutive days. Testing was performed in five consecutive sessions on the sixth and seventh days. For statistical analysis, we compared the means of the time until task disruption (latency to fall off the rotarod apparatus) over all ten runs.

#### 2.5.2. Open Field

For open field testing, animals were placed into the center of a dimly lit, opaque black box (50x50x40 cm). Animal movements were tracked by an automatic monitoring system (TSE Systems, Bad Homburg, Germany) for five minutes. Horizontal motor activity was evaluated by measuring travel distance. Anxiety was calculated as the percentage of time the animal stayed in the border areas of the box in relation to the total time spent in the maze (anxiety index, mean per animal). This procedure was repeated every 24 hours on four consecutive days in order to improve reliability and as training for the NOR [38]. Median value and interquartile range were calculated for each group. Vertical activity is given as mean rearing duration (i.e., standing on rear limbs) per treatment group ±SD. In addition to these variables, we recorded turning behavior with counter-clockwise movements and hyperactivity.

#### 2.5.3. Novel Object Recognition

Following open field, we performed NOR on consecutive days with a test-free interval of 48 hours. The NOR is designed as a two-trial, nonspatial, nonaversive memory test, consisting of a sample phase and a “choice” phase interrupted by a defined free interval (30 minutes, 5 hours, and 24 hours) during which the animals were returned to their cages. In the “choice” phase, one of the objects was replaced by a new one and the exploration was repeated for five minutes. They were placed at a distance (distance to centre) of 14 cm to the wall and 15 cm from each other. Novel object recognition was assessed by means of an automatic video tracking system (TSE Systems, Bad Homburg, Germany), using “three-point detection,” which distinguishes between the head, rear end, and centre of gravity of the animal, allowing monitoring when the animal is exploring a specific object with its nose. An encounter was defined as sniffing or rearing on the object. The objects were designed in such a way that sitting on the object was impossible for the animals. Object recognition was defined as spending more time with the novel object, indicating ability to remember the familiar object. For better comparability in statistical analyses a discrimination index (DI) was calculated as follows:

(1) $$\begin{array}{c} DI=\frac{\left(tN-tF\right)}{\left(tN+tF\right)} \end{array}$$

where t_N_ is the time spent with the novel and t_F_ is the time spent with the familiar object [39]. Positive values indicate a preference for the novel, and negative values indicate a preference for the familiar object.

### 2.6. Statistics

Statistical analyses were performed with IBM SPSS Statistics, version 24 for Windows (SPSS Inc., Armonk, NY, USA) and SigmaPlot, version 12 (Systat Software, Erkrath, Germany). Qualitative data assessment was conducted with the Pearson Chi-Square test. Data distribution was evaluated by means of histogram analysis and the Shapiro-Wilk test. For comparisons between two groups, a Mann-Whitney U test was used if data were not normally distributed. If needed, correction for multiple testing was carried out by means of the Bonferroni method. For assessment of overall differences between multiple groups, one-way analysis of variance (ANOVA) with Tukey's post-hoc analysis was performed, if data were normally distributed. For analysis of data not belonging to a particular distribution, a Kruskal-Wallis test with subsequent nonparametric multiple comparison testing according to the Student-Newman-Keuls method was applied. Post hoc analyses between groups were only performed in cases where statistically significant overall differences were detected. For nonparametric correlations, Spearman's rho correlation coefficient was used. Significance level was set at p<0.05.

## 3. Results

### 3.1. Study Population

Baseline histopathological injury was evaluated in sham-treated animals (P10, n=10; P18, n=8); baseline neurobehavioral outcome was established in healthy, untreated control animals (P90, n=5). For the evaluation of treatment effects in HHI, a total number of 149 animals were included in the study; 114 animals survived until respective endpoint analyses (male: n=61, female: n=53). Of the 35 deceased animals, one died during anesthesia, eight during or immediately after hypoxia, seven due to maternal cannibalism at any point, eight between postnatal days 6 (P6) and 10, eight between P10 and P18, and two were late deaths (>P18). One animal died during behavioral testing (brain not harvested). In animals who did receive treatment, occurrence (*χ*^2^=0.163, p=0.922, Pearson Chi-Square, two degrees of freedom) and timing of death (*χ*^2^=4.911, p=0.297, Pearson Chi-Square, four degrees of freedom) did not significantly differ between treatment groups. None of the animals reached humane endpoint criteria with the need to be euthanised.

### 3.2. Brain Injury Assessment

**Figure 1:**
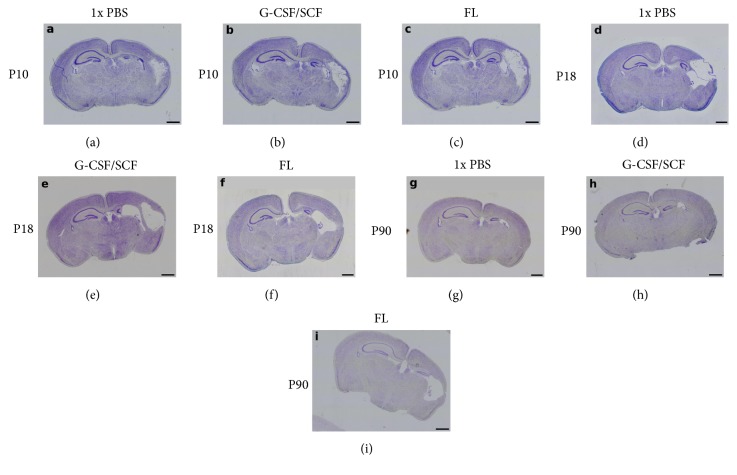
*Gross Brain Injury Assessment.* Representative images of injury extent in animals treated with 1x phosphate-buffered saline (PBS) (a, d, g), granulocyte colony-stimulating factor (G-CSF)/stem cell factor (SCF) (b, e, h), or Fms-related tyrosine kinase 3 ligand (FL) (c, f, i) for five consecutive days starting 60 hours after hypoxic-hyperoxic ischemic injury induction on postnatal day 5. Gross injury was assessed on postnatal days 10 (P10; (a)-(c)), 18 (P18; (d)-(f)), and 90 onwards (P90; (g)-(i)). Whole-brain visualisation was performed using a fourfold magnification. Brains were serially sectioned and stained with cresyl violet. Note the significant damage in the hemisphere ipsilateral to the right ligated common carotid artery. Scale bar indicates 1000 *μ*m.

Baseline median gross brain injury in sham-treated animals was 0.00 (minimum 0.00, maximum 0.00) at all evaluated time points. In HHI, gross brain injury did not differ between treatment groups at any time point (P10: H=2.779, p=0.249, n=41; P18: H=2.256, p=0.324, n=39; P90: H=1.966, p=0.374, n=34; Kruskal-Wallis test, two degrees of freedom). Details can be found in Table 1, representative images of cresyl violet staining for gross brain injury assessment are shown in Figure 1. No sex-specific differences were observed (Mann-Whitney U test with Bonferroni correction for multiple tests; details are provided in Supplementary Table 1).

Baseline median neuropathological injury scores in sham-treated animals were 0.0 in all brain regions examined at all time points (all minimum 0.0, maximum 0.0; except for P10, white matter: maximum 0.5; P18, cerebral cortex: maximum 0.5).

In HHI, neuropathological injury scores did not significantly differ between treatment groups at any time point in cerebral cortex (P10: H=2.741, p=0.254; P18: H=2.497, p=0.287; P90: H=2.677, p=0.262; Kruskal-Wallis test, two degrees of freedom), adjacent white matter (P10: H=1.279, p=0.527; P18: H=0.528, p=0.768; P90: H=2.610, p=0.271; Kruskal-Wallis test, two degrees of freedom), hippocampus (P10: H=0.982, p=0.612; P18: H=0.894, p=0.639; P90: H=1.338, p=0.512; Kruskal-Wallis test, two degrees of freedom), thalamus (P10: H=0.700, p=0.705; P18: H=0.115, p=0.944; P90: H=1.779, p=0.411; Kruskal-Wallis test, two degrees of freedom), or striatum (P10: H=4.132, p=0.127; P18: H=0.688, p=0.709; P90: H=3.487, p=0.175; Kruskal-Wallis test, two degrees of freedom). Details can be found in Table 2. No sex-specific differences were observed (Mann-Whitney U test with Bonferroni correction for multiple tests).

### 3.3. Caspase-3-Mediated Apoptosis

**Figure 2:**
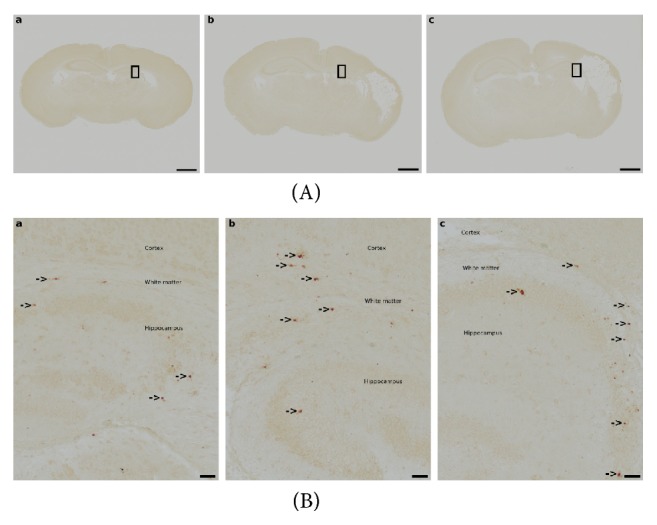
*Caspase-3-Mediated Apoptosis.* Representative images of activated caspase-3 positive cells in animals treated with 1x phosphate-buffered saline (PBS) (a), granulocyte colony-stimulating factor (G-CSF)/stem cell factor (SCF) (b), or Fms-related tyrosine kinase 3 ligand (FL) (c) for five consecutive days starting 60 hours after hypoxic-hyperoxic ischemic injury induction on postnatal day 5. Caspase-3-mediated apoptosis was assessed on postnatal day 10. Whole-brain visualisation was performed using a fourfold magnification (scale bar = 1000 *μ*m) (A). Regions of interest encompassing grey matter, white matter, and hippocampal structures are indicated by rectangles in (A) a-c and were magnified using a tenfold magnification (scale bar = 50 *μ*m) (B). Arrows indicate activated caspase-3-positive cells.

On P10, the number of activated caspase-3-positive cells did not significantly differ between treatment groups in any brain region in the ipsilateral hemisphere (grey matter: H=0.413, p=0.813; white matter: H=0.863, p=0.649; hippocampus: H=0.404, p=0.817; striatum: H=1.013, p=0.603; thalamus: H=3.406, p=0.182; n=16-17, Kruskal-Wallis test, two degrees of freedom) and in the contralateral hemisphere (grey matter: H=0.281, p=0.869; white matter: H=2.338, p=0.311; hippocampus: H=0.154, p=0.926; striatum: H=0.299, p=0.861; thalamus: H=1.427, p=0.490; n=16-17, Kruskal-Wallis test, two degrees of freedom). Details are presented in Table 3, and representative images of activated caspase-3 positive cells are shown in Figure 2. The number of activated caspase-3-positive cells in any of the regions examined was not significantly correlated with gross brain injury (Spearman rho correlation coefficient, all p>0.05, all data available on request).

With regard to sex, no significant differences were detected between male and female experimental animals in all treatment groups (Mann-Whitney U test with Bonferroni correction for multiple tests; details are displayed in Supplementary Figure 1).

### 3.4. Caspase-Independent Apoptosis

**Figure 3:**
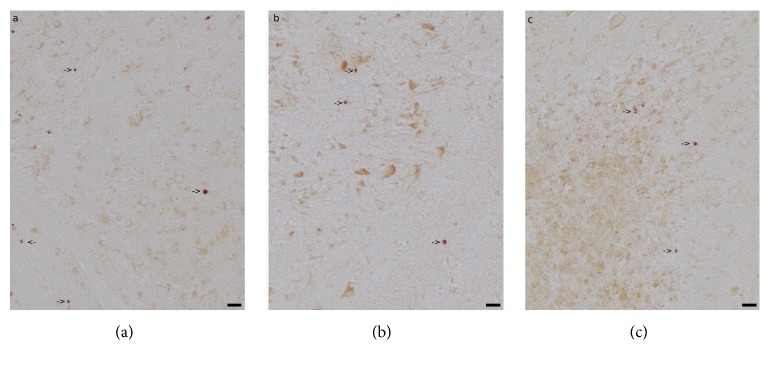
*Caspase-Independent Apoptosis.* Representative images of apoptosis-inducing factor- (AIF-) positive nuclei in animals treated with 1x phosphate-buffered saline (PBS) (a), granulocyte colony-stimulating factor (G-CSF)/stem cell factor (SCF) (b), or Fms-related tyrosine kinase 3 ligand (FL) (c) for five consecutive days starting 60 hours after hypoxic-hyperoxic ischemic injury induction on postnatal day 5. Caspase-independent apoptosis was assessed by AIF positivity on postnatal day 10. Images represent 20-fold magnification (scale bar = 20 *μ*m). Arrows indicate AIF-positive nuclei in grey matter structures.

On P10, the number of AIF-positive nuclei did not significantly differ between treatment groups in any brain region in the ipsilateral hemisphere (grey matter: H=0.092, p=0.955; white matter: H=0.701, p=0.704; hippocampus: H=0.202, p=0.904; striatum: H=1.185, p=0.553; thalamus: H=5.401, p=0.067; n=9, Kruskal-Wallis test, two degrees of freedom) or in the contralateral hemisphere (grey matter: H=0.638, p=0.727; white matter: H=1.708, p=0.426; hippocampus: H=0.836, p=0.658; striatum: H=2.279, p=0.320; thalamus: H=2.508, p=0.285; n=9, Kruskal-Wallis test, two degrees of freedom). Details are presented in Table 4, and representative images of AIF-staining are shown in Figure 3. No sex-specific differences were detected (Mann-Whitney U test with Bonferroni correction for multiple tests, all p>0.05).

### 3.5. Cell Proliferation

On P18, statistical analysis revealed an overall difference in the number of 5-bromo-2′-deoxyuridine- (BrdU-) positive cells in the granular layer of the hippocampal dentate gyrus in the ipsilateral hemisphere (H=6.038, p=0.049; n=17; Kruskal-Wallis test, two degrees of freedom). However, significance was lost in pairwise comparisons (1x PBS vs. G-CSF/SCF: p=0.050; 1x PBS vs. FL: p=0.738; Student-Newman-Keuls method). The number of BrdU-positive cells in other brain regions examined in the ipsilateral hemisphere did not significantly differ between treatment groups (grey matter: H=0.573, p=0.751; white matter: H=1.740, p=0.419; field CA1 of the hippocampus: H=0.966, p=0.617; subventricular zone: H=0.325, p=0.850; n=14-18, Kruskal-Wallis test, two degrees of freedom). No overall differences in the number of BrdU-positive cells were detected in the contralateral hemisphere (grey matter: H=0.784, p=0.676; white matter: H=2.327, p=0.312; granular layer of the hippocampal dentate gyrus: H=0.433, p=0.805; field CA1 of the hippocampus: H=0.292, p=0.864; subventricular zone: H=2.433, p=0.296; n=18, Kruskal-Wallis test, two degrees of freedom).

**Figure 4:**
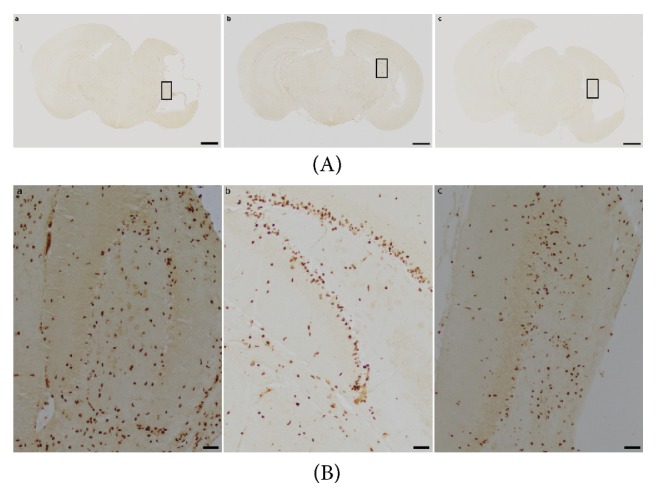
*Cell Proliferation.* Representative images of BrdU-positive cells in animals treated with 1x phosphate-buffered saline (PBS) (a), granulocyte colony-stimulating factor (G-CSF)/stem cell factor (SCF) (b), or Fms-related tyrosine kinase 3 ligand (FL) (c) for five consecutive days starting 60 hours after hypoxic-hyperoxic ischemic injury induction. Whole-brain visualisation was performed using a fourfold magnification (scale bar = 1000 *μ*m) (A). Hippocampal dentate gyrus was visualised using a tenfold magnification (scale bar = 50 *μ*m) (B).

On P90, the number of BrdU-positive cells did not significantly differ between treatment groups in any brain region in the ipsilateral hemisphere (grey matter: H=4.209, p=0.122; white matter: H=4.288, p=0.117; granular layer of the hippocampal dentate gyrus: H=2.721, p=0.257; field CA1 of the hippocampus: H=3.722, p=0.156; subventricular zone: H=0.069, p=0.966; n=12-21, Kruskal-Wallis test, two degrees of freedom) or contralateral hemisphere (grey matter: H=1.539, p=0.463; white matter: H=2.271, p=0.321; granular layer of the hippocampal dentate gyrus: H=1.399, p=0.497; field CA1 of the hippocampus: H=1.610, p=0.447; subventricular zone: H=1.124, p=0.570; n=19-21, Kruskal-Wallis test, two degrees of freedom). Details are given in Tables 5 and 6, and data represent absolute cell numbers in the respective brain areas. Representative images of BrdU-positive cells are shown in Figure 4.

On P18 and P90, the number of BrdU-positive vessels in the various treatment groups showed no significant difference in grey and white matter in the ipsilateral hemisphere (P18: grey matter: H=1.310, p=0.519; white matter: H=1.077, p=0.584; n=17-18; P90: grey matter: H=3.939, p=0.140; white matter: H=1.939, p=0.379; n=20; Kruskal-Wallis test, two degrees of freedom) or contralateral hemisphere (P18: grey matter: H=2.485, p=0.289; white matter: H=1.206, p=0.547; n=18; P90: grey matter: H=2.613, p=0.271; white matter: H=1.976, p=0.372; n=20; Kruskal-Wallis test, two degrees of freedom). Details are shown in Table 7. No statistically significant sex-differences in cell proliferation or the number of BrdU-positive vessels were detected neither on P18 nor on P90 in any region evaluated. Details are depicted in Supplementary Figures 2 and 3.

### 3.6. Functional Outcome

On P90, body weight did not significantly differ between treatment groups in animals subjected to HHI (mean bodyweight (standard deviation, SD) [g]; 1x PBS: 37.1 (11.1), G-CSF/SCF: 37.3 (5.8), FL: 34.6 (6.3); n=11-12 per group, F=0.377, p=0.689, One-Way ANOVA, two degrees of freedom). Female mice were significantly lighter in 1x PBS and G-CSF/SCF treatment groups, whereas body weight did not differ between male and female experimental animals in FL-treated mice (mean bodyweight (SD) [g]; 1x PBS: male 44.6 (9.6), female 28.0 (2.7), n=5-6, t=3.727, p= 0.005, Student's t-test; G-CSF/SCF: male 41.1 (3.8), female 31.9 (2.8), n=5-7, t=4.528, p=0.001, Student's t-test; FL: male 35.5 (7.2), female 33.0 (5.0), n=4-7, t=0.618, p=0.552, Student's t-test; Bonferroni-adjusted level of significance p<0.025).

**Figure 5:**
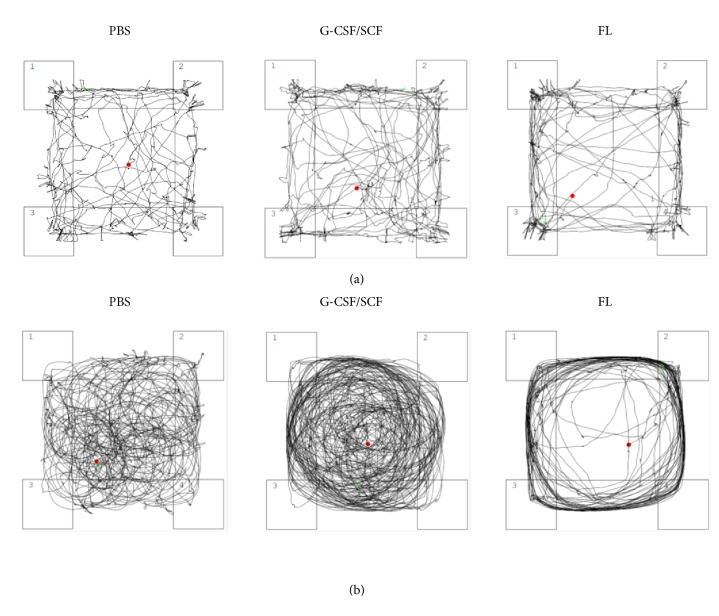
*Open Field*. Representative depiction of normal (a) and counter-clockwise movements and hyperactivity (“turning behavior”) (b) in 90-day-old CD-1 mice subjected to hypoxic-hyperoxic ischemic brain injury on postnatal day 5 receiving daily treatment with 1x phosphate-buffered saline (PBS), granulocyte colony-stimulating factor (G-CSF)/stem cell factor (SCF), or Fms-related tyrosine kinase 3 ligand (FL) for five consecutive days starting 60 hours after injury.

Turning behavior with counter-clockwise movements and hyperactivity was not observed in healthy control animals, but it occurred in six animals subjected to HHI, indicating severe brain damage (1x PBS: n=1 (9.1%) (female), G-SCF/SCF: n=2 (16.7%) (1 male, 1 female), FL: n=3 (27.3%) (2 male, 1 female); *χ*^2^=1.263, p=0.532, Pearson Chi-Square, two degrees of freedom; Figure 5). As the assessment of neurobehavioral endpoints with accelerod, open field, or novel object recognition testing is not possible in these animals, they were excluded from further behavioral analyses.

With regard to accelerod performance, baseline time until task disruption in healthy control animals was 97.2 ± 46 sec (mean ± SD).

In HHI, accelerod performance did not significantly differ between treatment groups (mean time until task disruption (SD) [sec]; 1x PBS: 107.7 (46.7), G-CSF/SCF: 116.3 (35.6), FL: 146.7 (74.4); n=11-12 per group, F=1.624, p=0.213, One-Way ANOVA, two degrees of freedom). No statistically significant sex-specific differences were detected (n=5-7, Student's t-test with Bonferroni-adjusted level of significance).

With regard to open field assessments, baseline anxiety index in sham-treated animals was 80.6% (76.9, 91.5) (median (IQR)), baseline travel distance as an indicator of horizontal activity was 3000.3 cm (2465.2, 3264.9) (median (IQR)), and baseline time spent rearing as an indicator of vertical activity was 68.1 sec (12.9) (mean (SD)).

In HHI, anxiety (median anxiety index (IQR) [%]; 1x PBS: 85.0 (75.0, 90.1), G-CSF/SCF: 78.8 (71.8, 83.2), FL: 85.4 (77.8, 87.5); n=34, H=2.291, p=0.318, Kruskal-Wallis test, two degrees of freedom), horizontal activity (median travel distance (IQR) [cm]; 1x PBS: 2871.6 (2259.9, 3175.0); G-CSF/SCF: 3132.2 (2934.5, 3660.6), FL: 3420.1 (2582.9, 11268.5); n=34, H=4.191, p=0.123, Kruskal-Wallis test, two degrees of freedom), and vertical activity (mean time spent rearing (SD) [sec]; 1x PBS: 71.6 (29.4), G-SCF/SCF: 85.0 (33.4), FL: 68.7 (49.3); n=11-12 per group, F=0.603, p=0.554, One-Way ANOVA, two degrees of freedom) did not significantly differ between treatment groups. No statistically significant sex-specific differences were detected (n=5-7, Student's t-test with Bonferroni-adjusted level of significance).

With regard to novel object recognition, baseline discrimination index (DI) in sham-treated animals was 0.16 (0.01, 0.28) (median (IQR)) after one hour, 0.29 (-0.15, 0.54) (median (IQR)) after three hours, and 0.35 (0.12, 0.48) (median (IQR)) after five hours.

**Figure 6:**
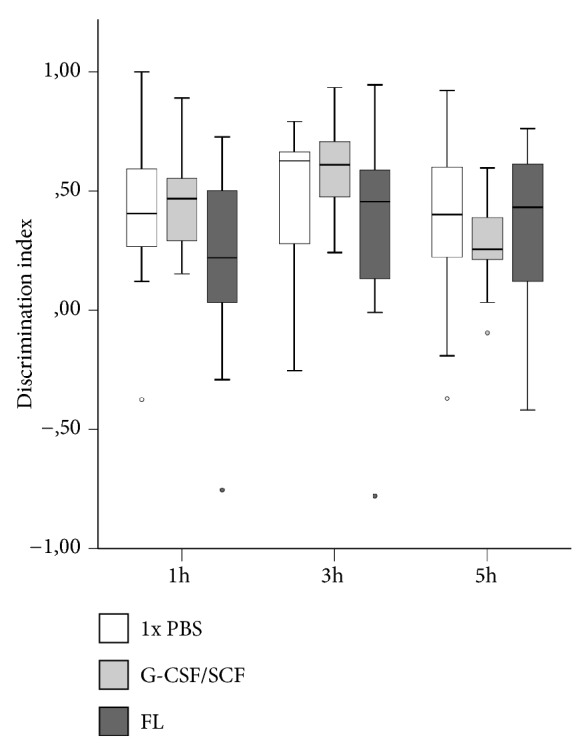
Long-term behavioral assessment following hypoxic-hyperoxic ischemic brain injury in CD-1 mice on postnatal day 90 by means of novel object recognition testing. Experiments were performed on three consecutive days. Mice were first placed in a dimly lit, opaque black box containing two identical objects for five minutes and were then returned to their cages. After a defined interval (one hour (1 h) on day 1, three hours (3 h) on day 2, and five hours (5 h) on day 3) they were put back in the apparatus, which now contained one familiar and one novel object. The time spent at each object was recorded and a discrimination index (DI) was calculated as follows: DI=((t_N_-t_F_))/((t_N_+t_F_)), where t_N_ is the time spent with the novel and t_F_ is the time spent with the familiar object. Positive values indicate a preference for the novel, and negative values indicate a preference for the familiar object. No statistically significant differences in DI were observed between treatment groups (1 h: H=2.269, p=0.322; 3 h: H=1.942, p=0.379; 5 h: H=1.250, p=0.535; n=34, Kruskal-Wallis test, two degrees of freedom). The dark lines in the middle of each box represent median values, box edges represent 25^th^ and 75^th^ percentiles, whiskers correspond to 1.5-fold interquartile ranges, and dots represent outliers (FL, Fms-related tyrosine kinase 3 ligand; G-CSF/SCF, granulocyte colony-stimulating factor/stem cell factor; PBS, phosphate-buffered saline).

In HHI, no overall differences in DI between treatment groups were observed after one (median DI (IQR); 1x PBS: 0.41 (0.18, 0.60), G-CSF/SCF: 0.47 (0.26, 0.58), FL: 0.22 (-0.11, 0.50); n=34, H=2.269, p=0.322, Kruskal-Wallis test, two degrees of freedom), three (median DI (IQR); 1x PBS: 0.63 (0.12, 0.66), G-CSF/SCF: 0.61 (0.45, 0.72), FL: 0.46 (0.00, 0.61); n=34, H=1.942, p=0.379, Kruskal-Wallis test, two degrees of freedom), or five hours (median DI (IQR); 1x PBS: 0.40 (0.15, 0.67), G-CSF/SCF: 0.26 (0.21, 0.41), FL: 0.43 (0.03, 0.63); n=34, H=1.250, p=0.535, Kruskal-Wallis test, two degrees of freedom). Details can be seen in Figure 6. No statistically significant sex-specific differences were detected (n=5-7, Student's t-test with Bonferroni-adjusted level of significance).

## 4. Discussion

The identification of novel treatment options for brain injury is one of the biggest challenges encountered in neonatal neuroscience [13, 14, 40, 41]. Various cytokines and hematopoietic growth factors have been shown to have tissue-protective properties independent of their hematologic effects and have been investigated as therapeutic strategies in neonatal brain injury [42]. Several of them have yielded promising results in preclinical trials. However, due to the pleiotropic nature of many of these agents and their context-specific mechanisms of action, meticulous assessment of their safety profiles is essential prior to potential routine clinical use [5, 12]. Our research group has previously shown that the hematopoietic growth factors G-CSF/SCF and FL can exert both beneficial and injurious effects on neonatal brain injury, depending on type of injury and timing of treatment [29–31]. To the best of our knowledge their short-, mid-, and long-term effects in multiple-stressor neonatal brain injury models have not yet been systematically evaluated.

In the study at hand, we used a combined hypoxic-hyperoxic ischemic brain injury model, thought to more adequately reflect the clinical situation in neonatal intensive care units [32, 34]. Also, in this model, hypoxia-related mortality is regarded to be less pronounced, while brain injury can still be reliably induced [34]. In our study, the mortality rate was 23.5% and thus higher than previously described [34]. However, this finding did not reach statistical significance (p=0.090, Fisher's Exact test). Of relevance, mortality was not influenced by treatment type.

On a histomorphological basis, we did not observe statistically significant differences between treatment groups at any time point, either in gross brain injury, or in caspase-dependent or -independent apoptosis as indicated by AIF-positivity. Another mechanism of action described for G-CSF/SCF is its trophic effect on neurons [28]. An increase in cell proliferation could be mediated by an enhanced release of CD34+ cells due to G-CSF/SCF application [30], which has been shown to increase angio- and neurogenesis and to improve morphological and functional recovery [43]. For this reason, we investigated whether G-CSF/SCF and FL treatment increases cell proliferation and the number of BrdU-positive vessels at the lesion site but did not observe a statistically significant difference.

This is in discordance with previous neonatal rodent brain injury studies, reporting reduced damage extent, apoptotic cell death, and increased cell proliferation following both single and repetitive G-CSF treatments [18, 19, 21, 23, 24, 28]. Also, in our study functional outcomes did not significantly differ between treatment groups—in contrast to previously published works reporting improvement in long-term cognitive and motor functions after G-CSF and SCF treatment [20, 22, 25, 27, 28]. However, all aforementioned experiments were conducted in newborn rat pups. As species and strain differences in brain injury susceptibility have been reported, a differential response to treatments in mice is entirely possible [44, 45]. Of relevance, our own research group has shown no improvement in brain injury extent or neurobehavioral outcome following G-CSF treatment in newborn mice subjected to a modified Rice-Vannucci procedure [29]. In general, treatment effects might depend on type of injury, as all aforementioned studies used hypoxia-ischemia [18–25, 29] or hypoxia only [27, 28] for injury induction. In a mouse model of neonatal excitotoxic brain injury, we previously observed an exacerbation of grey and white matter damage by G-CSF/SCF when administered in the acute phase of injury [30]. When administered 60 hours after injury, however, G-CSF/SCF and FL treatments led to a significant reduction in lesion size and a decrease in caspase-3 activation in the same injury model [31]. The very same treatment regimina were applied in the current study. As we have shown that the administration of substances or even vehicle solutions in the acute phase of injury might lead to increased brain damage in newborn mice [29, 30], an acute treatment strategy was not attempted again for animal-sparing reasons. This is also one of the main limitations of our study: We cannot rule out that the therapeutic window in combined hypoxic-hyperoxic ischemic neonatal brain injury might be narrow and an earlier intervention might be beneficial. However, bearing in mind our previous findings of an increased vulnerability in the acute phase of injury and the principles of the 3Rs in animal experimentation, a de novo assessment of acute treatment strategies in this animal model did not seem justifiable.

For the present study, a higher dose of G-CSF was selected. The dose of 200 *μ*g/g body weight was chosen, based on previous publications and our own studies, showing a dose response curve [29, 30]. The effects of G-CSF dosage might be important, as Taguchi et al. reported impairments in behavior with increasing dosages of G-CSF following cerebral ischemia [43] The difference in the results might reflect the experimental model used, age and rodent used, the therapeutic time window, and the dose administered. As to why the treatment options conveying neuroprotection in the excitotoxic injury model [31] had no effect on hypoxic-hyperoxic ischemic brain injury, we can only speculate that the pathomechanisms leading to cell death following hypoxia-hyperoxia ischemia might be more complex, or injury extent more pronounced due to systemic hypoxia and hyperoxia and thus less susceptible to rescue treatments in general. Pathomechanisms involved in hypoxic-hyperoxic neonatal brain injury include alterations in apoptotic, inflammatory, and angiogenetic pathways, all of which are also implicated in hypoxic-ischemic brain injury [34] A direct comparison of these two injury models regarding pathogenesis and, particularly, contribution of individual factors would be of high relevance but is outstanding to date. Further studies evaluating this aspect in depth are required.

Interestingly, turning behavior as an indicator of severe brain damage was more frequently observed in G-CSF/SCF- and FL-treated animals than in vehicle-treated controls. Even though the finding was not statistically significant, this might still be of relevance as turning behavior has been shown to correlate with histological damage and sensory motor deficit in rodents exposed to cerebral ischemia [46]. Cautious use of these substances might thus be warranted.

The importance of evaluating sex-specific differences has been proven by several studies, especially when considering the variable responses of male and female immature rodents to brain injury [36, 47, 48]. Hence, we also conducted a sex-specific evaluation, but no sex-specific differences were present.

## 5. Conclusion

To conclude, in this multistressor mouse model of hypoxia-hyperoxia ischemia we cannot confirm the protective effect of G-CSF/SCF and FL on neonatal brain injury previously reported. As adverse effects cannot be ruled out, meticulous assessment of these hematopoietic growth factors in extensive preclinical studies is mandated prior to potential clinical use.

## Figures and Tables

**Table 1:** Evaluation of gross brain injury.

Time point	Treatment	Number of animals	Gross brain injury score; median (IQR)
*P10*	1x PBS	15	1.00 (0.00, 3.00)
	G-CSF/SCF	13	2.00 (0.50, 3.50)
	FL	13	3.00 (1.00, 4.00)
*P18*	1x PBS	12	1.00 (0.25, 1.75)
	G-CSF/SCF	14	1.00 (1.00, 1.25)
	FL	13	1.00 (0.00, 1.00)
*P90*	1x PBS	11	2.00 (1.00, 4.00)
	G-CSF/SCF	12	1.50 (1.00, 2.00)
	FL	11	3.00 (1.00, 4.00)

Abbreviations and acronyms: FL, Fms-related tyrosine kinase 3 ligand; G-CSF/SCF, granulocyte colony-stimulating factor/stem cell factor; IQR, interquartile range; P10/18/90, postnatal days 10/18/90; PBS, phosphate-buffered saline.

**Table 2:** Evaluation of neuropathological brain injury extent by region.

Time point	Treatment	Sample size	Cortex^§^	WM^§^	HC^§^	Thalamus^§^	Striatum^§^
*P10*	1x PBS	15	0.6 (0.3, 2.6)	1.0 (0.1, 2.8)	2.6 (1.8, 3.0)	0.0 (0.0, 1.3)	0.1 (0.0, 2.0)
	G-CSF/SCF	13	2.4 (0.5, 3.6)	2.9 (0.5, 3.6)	2.9 (2.5, 3.0)	0.0 (0.0, 2.3)	2.0 (0.1, 2.9)
	FL	13	2.5 (0.5, 3.9)	2.8 (0.0, 4.0)	2.5 (1.8, 3.0)	0.0. (0.0, 3.0)	2.0 (0.1, 3.0)
*P18*	1x PBS	11	0.8 (0.4, 1.5)	0.9 (0.6, 2.3)	1.2 (1.0, 2.6)	0.5 (0.0, 1.3)	0.3 (0.0, 0.7)
	G-CSF/SCF	14	0.8 (0.6, 1.4)	0.8 (0.7, 1.7)	1.9 (1.0, 2.7)	0.7 (0.4, 1.0)	0.5 (0.0, 1.0)
	FL	12	0.6 (0.4, 1.2)	0.8 (0.6, 1.5)	1.3 (1.1, 1.9)	0.8 (0.0, 1.0)	0.0 (0.0, 0.9)
*P90*	1x PBS	11	1.6 (0.6, 3.7)	1.8 (1.0, 3.7)	2.8 (0.9, 3.0)	0.0 (0.0, 2.4)	0.8 (0.0, 2.6)
	G-CSF/SCF	12	1.3 (0.7, 2.3)	1.3 (0.7, 2.3)	2.6 (1.4, 2.9)	0.8 (0.2, 1.7)	0.6 (0.0, 1.1)
	FL	11	3.3 (0.9, 4.0)	3.5 (0.9, 4.0)	3.0 (1.6, 3.0)	1.8 (0.0, 3.0)	2.6 (0.5, 3.0)

^§^Values represent median scores and interquartile ranges by region.

Abbreviations and acronyms: FL, Fms-related tyrosine kinase 3 ligand; G-CSF/SCF, granulocyte colony-stimulating factor/stem cell factor; HC, hippocampus; P10/18/90, postnatal days 10/18/90; PBS, phosphate-buffered saline; WM, white matter.

**Table 3:** Region-specific caspase-3 activation on postnatal day 10 (P10).

Region	Hemisphere	Treatment	Sample size	Caspase-3-positive cells; median (IQR)
*Grey matter*	*ipsilateral*	1x PBS	6	22 (21, 32)
		G-CSF/SCF	5	28 (22, 41)
		FL	6	25 (18, 40)
*Grey matter*	*contralateral*	1x PBS	6	20 (18, 22)
		G-CSF/SCF	5	20 (16, 22)
		FL	6	19 (16, 27)
*White matter*	*ipsilateral*	1x PBS	6	10 (7, 10)
		G-CSF/SCF	5	10 (9, 11)
		FL	6	9 (7, 10)
*White matter*	*contralateral*	1x PBS	6	8 (6, 10)
		G-CSF/SCF	5	7 (5, 9)
		FL	6	9 (7, 10)
*Hippocampus*	*ipsilateral*	1x PBS	6	15 (12, 24)
		G-CSF/SCF	5	14 (12, 23)
		FL	5	13 (9, 20)
*Hippocampus*	*contralateral*	1x PBS	6	9 (8, 16)
		G-CSF/SCF	5	11 (7, 12)
		FL	5	8 (7, 21)
*Striatum*	*ipsilateral*	1x PBS	6	9 (7, 14)
		G-CSF/SCF	5	11 (9, 15)
		FL	6	11 (8, 20)
*Striatum*	*contralateral*	1x PBS	6	8 (5, 9)
		G-CSF/SCF	5	7 (5, 9)
		FL	6	7 (6, 8)
*Thalamus*	*ipsilateral*	1x PBS	6	9 (7, 18)
		G-CSF/SCF	5	13 (8, 37)
		FL	5	20 (13, 38)
*Thalamus*	*contralateral*	1x PBS	6	8 (6, 11)
		G-CSF/SCF	5	9 (5, 11)
		FL	5	10 (8, 11)

Abbreviations and acronyms:FL, Fms-related tyrosine kinase 3 ligand; G-CSF/SCF, granulocyte colony-stimulating factor/stem cell factor; IQR, interquartile range; PBS, phosphate-buffered saline.

**Table 4:** Region-specific apoptosis-inducing factor (AIF) positivity on postnatal day 10 (P10).

Region	Hemisphere	Treatment	Sample size	AIF-positive nuclei; median (IQR)
*Grey matter*	*ipsilateral*	1x PBS	3	5 (5, 5)
		G-CSF/SCF	3	5 (5, 6)
		FL	3	5 (4, 6)
*Grey matter*	*contralateral*	1x PBS	3	4 (3, 4)
		G-CSF/SCF	3	4 (3, 5)
		FL	3	2 (2, 4)
*White matter*	*ipsilateral*	1x PBS	3	2 (1, 3)
		G-CSF/SCF	3	3 (2, 3)
		FL	3	1 (1, 2)
*White matter*	*contralateral*	1x PBS	3	1 (1, 2)
		G-CSF/SCF	3	2 (2, 2)
		FL	3	1 (1, 1)
*Hippocampus*	*ipsilateral*	1x PBS	3	4 (3, 5)
		G-CSF/SCF	3	4 (3, 4)
		FL	3	4 (4, 5)
*Hippocampus*	*contralateral*	1x PBS	3	1 (1, 1)
		G-CSF/SCF	3	1 (1, 1)
		FL	3	1 (1, 2)
*Striatum*	*ipsilateral*	1x PBS	3	2 (2, 2)
		G-CSF/SCF	3	1 (1, 2)
		FL	3	1 (1, 2)
*Striatum*	*contralateral*	1x PBS	3	1 (1, 2)
		G-CSF/SCF	3	1 (0, 1)
		FL	3	1 (1, 2)
*Thalamus*	*ipsilateral*	1x PBS	3	4 (4, 5)
		G-CSF/SCF	3	3 (3, 3)
		FL	3	7 (6, 7)
*Thalamus*	*contralateral*	1x PBS	3	2 (1, 2)
		G-CSF/SCF	3	3 (2, 3)
		FL	3	2 (2, 5)

Abbreviations and acronyms:FL, Fms-related tyrosine kinase 3 ligand; G-CSF/SCF, granulocyte colony-stimulating factor/stem cell factor; IQR, interquartile range; PBS, phosphate-buffered saline.

**Table 5:** Region-specific assessment of BrdU-positive cells on postnatal day 18 (P18).

Region	Hemisphere	Treatment	Sample size	BrdU-positive cells; median (IQR)
*Grey matter*	*ipsilateral*	1x PBS	6	558 (374, 690)
		G-CSF/SCF	6	513 (383, 621)
		FL	6	446 (387, 559)
*Grey matter*	*contralateral*	1x PBS	6	555 (350, 671)
		G-CSF/SCF	6	500 (430, 740)
		FL	6	460 (380, 581)
*White matter*	*ipsilateral*	1x PBS	5	412 (292, 518)
		G-CSF/SCF	6	295 (261, 436)
		FL	6	342 (278, 431)
*White matter*	*contralateral*	1x PBS	6	407 (289, 522)
		G-CSF/SCF	6	351 (296, 399)
		FL	6	296 (230, 410)
*GrDG*	*ipsilateral*	1x PBS	5	114 (98, 183)
		G-CSF/SCF	6	220 (143, 231)
		FL	6	127 (90, 155)
*GrDG*	*contralateral*	1x PBS	6	161 (107, 192)
		G-CSF/SCF	6	169 (130, 226)
		FL	6	174 (105, 225)
*CA1*	*ipsilateral*	1x PBS	5	52 (32, 71)
		G-CSF/SCF	5	57 (49, 96)
		FL	4	61 (42, 71)
*CA1*	*contralateral*	1x PBS	6	83 (39, 119)
		G-CSF/SCF	6	83 (62, 98)
		FL	6	94 (46, 118)
*SVZ*	*ipsilateral*	1x PBS	6	202 (150, 240)
		G-CSF/SCF	6	197 (178, 258)
		FL	6	174 (162, 236)
*SVZ*	*contralateral*	1x PBS	6	212 (150, 358)
		G-CSF/SCF	6	195 (168, 222)
		FL	6	164 (146, 194)

Abbreviations and acronyms:CA1, field CA1 of the hippocampus; FL, Fms-related tyrosine kinase 3 ligand; G-CSF/SCF, granulocyte colony-stimulating factor/stem cell factor; GrDG, granular layer of the hippocampal dentate gyrus; IQR, interquartile range; PBS, phosphate-buffered saline; SVZ, subventricular zone.

**Table 6:** Region-specific assessment of BrdU-positive cells on postnatal day 90 (P90).

Region	Hemisphere	Treatment	Sample size	BrdU-positive cells; median (IQR)
*Grey matter*	*ipsilateral*	1x PBS	7	152 (101, 309)
		G-CSF/SCF	7	281 (263, 383)
		FL	6	315 (202, 408)
*Grey matter*	*contralateral*	1x PBS	7	305 (115, 432)
		G-CSF/SCF	7	303 (226, 389)
		FL	6	357 (324, 462)
*White matter*	*ipsilateral*	1x PBS	7	53 (30, 180)
		G-CSF/SCF	7	225 (92, 235)
		FL	6	144 (55, 285)
*White matter*	*contralateral*	1x PBS	7	216 (54, 244)
		G-CSF/SCF	7	263 (138, 306)
		FL	6	250 (212, 278)
*GrDG*	*ipsilateral*	1x PBS	6	34 (17, 59)
		G-CSF/SCF	6	68 (33, 90)
		FL	4	65 (40, 76)
*GrDG*	*contralateral*	1x PBS	6	75 (42, 105)
		G-CSF/SCF	6	85 (72, 101)
		FL	6	72 (62, 86)
*CA1*	*ipsilateral*	1x PBS	4	18 (4, 65)
		G-CSF/SCF	5	69 (24, 125)
		FL	3	92 (64, 110)
*CA1*	*contralateral*	1x PBS	7	62 (12, 93)
		G-CSF/SCF	7	82 (51, 103)
		FL	6	73 (29, 77)
*SVZ*	*ipsilateral*	1x PBS	7	7 (5, 15)
		G-CSF/SCF	7	7 (5, 9)
		FL	7	6 (6, 11)
*SVZ*	*contralateral*	1x PBS	7	7 (2, 8)
		G-CSF/SCF	7	5 (4, 10)
		FL	7	8 (7, 9)

Abbreviations and acronyms:CA1, field CA1 of the hippocampus; FL, Fms-related tyrosine kinase 3 ligand; G-CSF/SCF, granulocyte colony-stimulating factor/stem cell factor; GrDG, granular layer of the hippocampal dentate gyrus; IQR, interquartile range; PBS, phosphate-buffered saline; SVZ, subventricular zone.

**Table 7:** Region-specific assessment of BrdU-positive vessels.

Day of life	Region	Hemisphere	Treatment	Sample size	BrdU-positive vessels; median (IQR)
*P18*	*Grey matter*	*ipsilateral*	1x PBS	6	23 (19, 35)
			G-CSF/SCF	6	30 (24, 41)
			FL	6	28 (24, 40)
	*Grey matter*	*contralateral*	1x PBS	6	22 (20, 35)
			G-CSF/SCF	6	30 (28, 37)
			FL	6	24 (20, 49)
	*White matter*	*ipsilateral*	1x PBS	5	5 (4, 10)
			G-CSF/SCF	6	7 (5, 9)
			FL	6	5 (3, 9)
	*White matter*	*contralateral*	1x PBS	6	8 (4, 9)
			G-CSF/SCF	6	11 (6, 14)
			FL	6	8 (5, 12)
*P90*	*Grey matter*	*ipsilateral*	1x PBS	7	23 (14, 42)
			G-CSF/SCF	7	45 (30, 77)
			FL	6	41 (29, 53)
	*Grey matter*	*contralateral*	1x PBS	7	47 (11, 56)
			G-CSF/SCF	7	62 (34, 84)
			FL	6	67 (48, 76)
	*White matter*	*ipsilateral*	1x PBS	7	1 (0, 14)
			G-CSF/SCF	7	5 (4, 12)
			FL	6	8 (1, 13)
	*White matter*	*contralateral*	1x PBS	7	9 (8, 12)
			G-CSF/SCF	7	10 (3, 13)
			FL	6	14 (8, 18)

Abbreviations and acronyms:FL, Fms-related tyrosine kinase 3 ligand; G-CSF/SCF, granulocyte colony-stimulating factor/stem cell factor; IQR, interquartile range; P18/90, postnatal days 18/90; PBS, phosphate-buffered saline.

## Data Availability

All data generated or analysed during this study are included in this published article (and its supplementary information files).

## Abbreviations

bw: Body weight
FL: Fms-related tyrosine kinase 3 ligand
G-CSF: Granulocyte colony-stimulating factor
HHI: Hypoxic-hyperoxic ischemic brain injury
Pxx: Postnatal day xx
SCF: Stem cell factor.

## Supplementary Materials

Supplementary Materials
* Supplementary Figure 1: gender-specific differences in caspase-3 activation on postnatal day 10 following hypoxic-hyperoxic brain injury*. M (open bars) represent male subjects, and F (hatched bars) represent female subjects. No gender-specific differences in caspase-3 activation were observed in any treatment group in any brain region assessed. Data are presented as median plus IQR. FL, Fms-related tyrosine kinase 3 ligand; G-CSF/SCF, granulocyte colony-stimulating factor/stem cell factor; IQR, interquartile range; PBS, phosphate-buffered saline.* Supplementary Figure 2: gender-specific differences in BrdU-positive cells on postnatal days 18 (A) and 90 (B) following hypoxic-hyperoxic brain injury*. M (open bars) represent male subjects, and F (hatched bars) represent female subjects. No gender-specific differences in caspase-3 activation were observed in any treatment group in any brain region assessed. Data are presented as median plus IQR. CA1, field CA1 of the hippocampus; FL, Fms-related tyrosine kinase 3 ligand; G-CSF/SCF, granulocyte colony-stimulating factor/stem cell factor; GrDG, granular layer of the hippocampal dentate gyrus; IQR, interquartile range; PBS, phosphate-buffered saline; SVZ, subventricular zone.* Supplementary Figure 3: gender-specific differences in BrdU-positive vessels on postnatal days 18 (A) and 90 (B) following hypoxic-hyperoxic brain injury*. M (open bars) represent male subjects, and F (hatched bars) represent female subjects. No gender-specific differences in caspase-3 activation were observed in any treatment group in any brain region assessed. Data are presented as median plus IQR. FL, Fms-related tyrosine kinase 3 ligand; G-CSF/SCF, granulocyte colony-stimulating factor/stem cell factor; IQR, interquartile range; PBS, phosphate-buffered saline.* Supplemental Table 1: evaluation of gross brain injury by sex*. Values represent median scores and interquartile ranges. Abbreviations and acronyms: FL, Fms-related tyrosine kinase 3 ligand; G-CSF/SCF, granulocyte colony-stimulating factor/stem cell factor; IQR, interquartile range; P10/18/90, postnatal days 10/18/90; PBS, phosphate-buffered saline.
