# Supplementary material for: Short-, Mid-, and Long-Term Effect of Granulocyte Colony-Stimulating Factor/Stem Cell Factor and Fms-Related Tyrosine Kinase 3 Ligand Evaluated in an In Vivo Model of Hypoxic-Hyperoxic Ischemic Neonatal Brain Injury
Source: Biomed Res Int. 2019 Mar 13;2019:5935279. doi: 10.1155/2019/5935279 (PMC6436372; doi:10.1155/2019/5935279)
Supplement: Supplementary Materials — Supplementary Figure 1: gender-specific differences in caspase-3 activation on postnatal day 10 following hypoxic-hyperoxic brain injury. M (open bars) represent male subjects, and F (hatched bars) represent female subjects. No gender-specific differences in caspase-3 activation were observed in any treatment group in any brain region assessed. Data are presented as median plus IQR. FL, Fms-related tyrosine kinase 3 ligand; G-CSF/SCF, granulocyte colony-stimulating factor/stem cell factor; IQR, interquartile range; PBS, phosphate-buffered saline. Supplementary Figure 2: gender-specific differences in BrdU-positive cells on postnatal days 18 (A) and 90 (B) following hypoxic-hyperoxic brain injury. M (open bars) represent male subjects, and F (hatched bars) represent female subjects. No gender-specific differences in caspase-3 activation were observed in any treatment group in any brain region assessed. Data are presented as median plus IQR. CA1, field CA1 of the hippocampus; FL, Fms-related tyrosine kinase 3 ligand; G-CSF/SCF, granulocyte colony-stimulating factor/stem cell factor; GrDG, granular layer of the hippocampal dentate gyrus; IQR, interquartile range; PBS, phosphate-buffered saline; SVZ, subventricular zone. Supplementary Figure 3: gender-specific differences in BrdU-positive vessels on postnatal days 18 (A) and 90 (B) following hypoxic-hyperoxic brain injury. M (open bars) represent male subjects, and F (hatched bars) represent female subjects. No gender-specific differences in caspase-3 activation were observed in any treatment group in any brain region assessed. Data are presented as median plus IQR. FL, Fms-related tyrosine kinase 3 ligand; G-CSF/SCF, granulocyte colony-stimulating factor/stem cell factor; IQR, interquartile range; PBS, phosphate-buffered saline. Supplemental Table 1: evaluation of gross brain injury by sex. Values represent median scores and interquartile ranges. Abbreviations and acronyms: FL, Fms-related tyrosine kinase 3 ligand; G-C [file 5935279.f1.zip › 5935279.f1/5935279_SupplDesc.docx]

**Supplementary Figure 1: Gender-specific differences in caspase-3 activation on postnatal day 10 following hypoxic-hyperoxic brain injury.**

M (open bars) represent male, F (hatched bars) represent female subjects. No gender-specific differences in caspase-3 activation were observed in any treatment group in any brain region assessed. Data are presented as median plus IQR. FL, Fms-related tyrosine kinase 3 ligand; G-CSF/SCF, granulocyte colony-stimulating factor/stem cell factor; IQR, interquartile range; PBS, phosphate-buffered saline.

**Supplementary Figure 2: Gender-specific differences in BrdU-positive cells on postnatal days 18 (A) and 90 (B) following hypoxic-hyperoxic brain injury.**

M (open bars) represent male, F (hatched bars) represent female subjects. No gender-specific differences in caspase-3 activation were observed in any treatment group in any brain region assessed. Data are presented as median plus IQR. CA1, field CA1 of the hippocampus; FL, Fms-related tyrosine kinase 3 ligand; G-CSF/SCF, granulocyte colony-stimulating factor/stem cell factor; GrDG, granular layer of the hippocampal dentate gyrus; IQR, interquartile range; PBS, phosphate-buffered saline; SVZ, subventricular zone.

**Supplementary Figure 3: Gender-specific differences in BrdU-positive vessels on postnatal days 18 (A) and 90 (B) following hypoxic-hyperoxic brain injury.**

M (open bars) represent male, F (hatched bars) represent female subjects. No gender-specific differences in caspase-3 activation were observed in any treatment group in any brain region assessed. Data are presented as median plus IQR. FL, Fms-related tyrosine kinase 3 ligand; G-CSF/SCF, granulocyte colony-stimulating factor/stem cell factor; IQR, interquartile range; PBS, phosphate-buffered saline.

***Supplemental Table 1: Evaluation of gross brain injury by sex***

Values represent median scores and interquartile ranges.

*Abbreviations and acronyms:* FL, Fms-related tyrosine kinase 3 ligand; G-CSF/SCF, granulocyte colony-stimulating factor/stem cell factor; IQR, interquartile range; P10/18/90, postnatal day 10/18/90; PBS, phosphate-buffered saline.
